# Three-dimensional gait analysis in spinal disorders: biomechanical insights and clinical applications for diagnosis, surgical planning, and rehabilitation

**DOI:** 10.3389/fneur.2025.1666267

**Published:** 2025-10-07

**Authors:** Jun Yin, Wei Cong, Yanguo Wang, Chao Zhou

**Affiliations:** Department of Spinal Surgery, Qilu Hospital of Shandong University (Qingdao), Qingdao, China

**Keywords:** three-dimensional gait analysis, spinal disorders, rehabilitation, central nervous system injury, cognitive impairment

## Abstract

Three-dimensional gait analysis technology offers a novel perspective for the study and clinical application of spinal disorders, enabling a deeper understanding of patients’ movement patterns and their biomechanical characteristics. This review synthesizes the use of three-dimensional gait analysis in spinal disorders, emphasizing its significance in diagnosis, surgical planning, and rehabilitation. By analyzing relevant literature, we explore how three-dimensional gait analysis assists in identifying biomechanical abnormalities associated with spinal diseases, optimizing surgical strategies, and enhancing rehabilitation outcomes. Furthermore, this article discusses future research directions and the potential impact of technological advancements on clinical practice, highlighting the essential role that gait analysis can play in improving patient care in the context of spinal disorders.

## 1 Introduction

**Figure 1:**
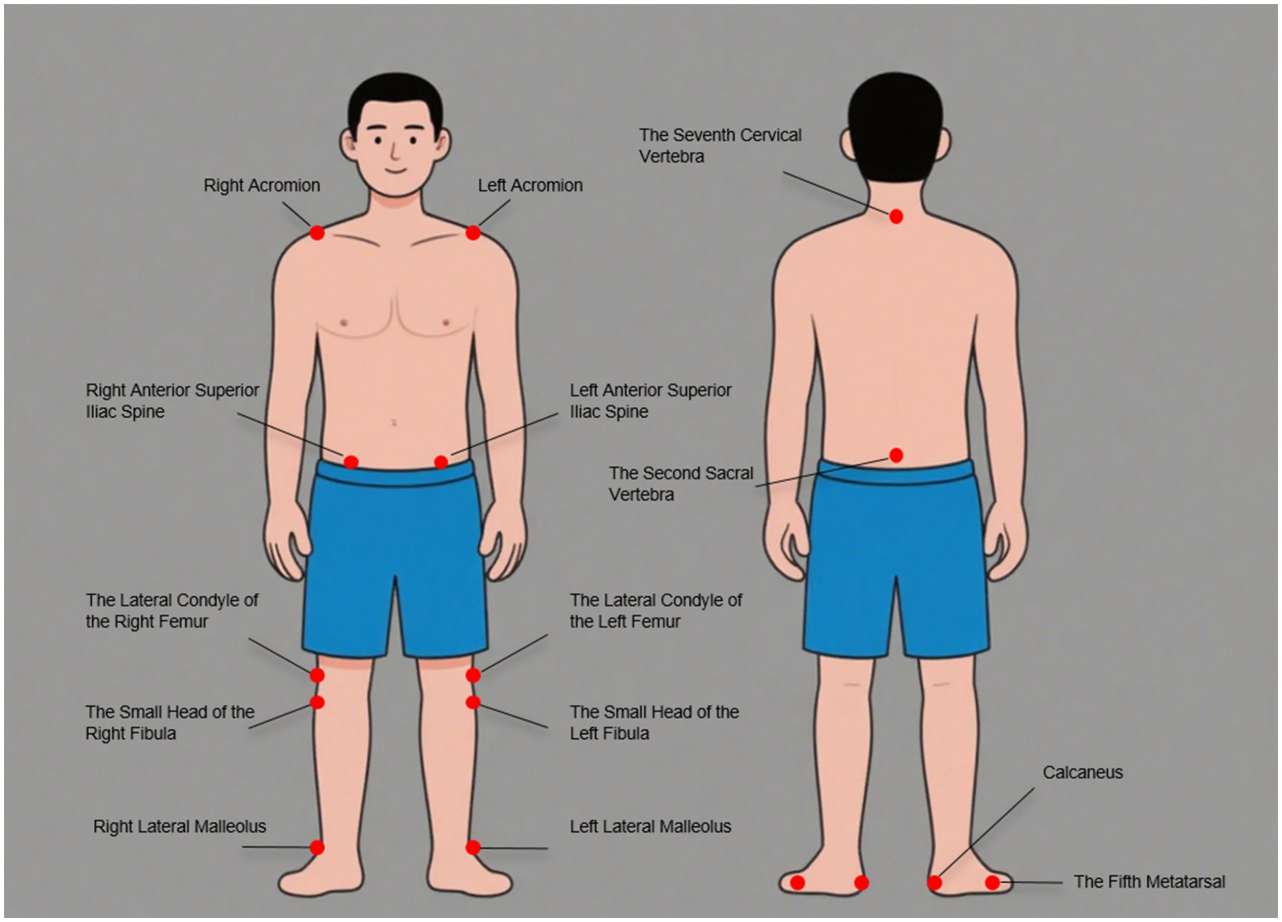
The anatomical positions where markers need to be placed for 3D gait analysis.

Human gait, defined as reciprocal bipedal locomotion, not only shapes an individual’s physical mobility but also fundamentally influences their social participation and interactive dynamics within societal frameworks, thereby underscoring its critical role in both motor function and human behavioral engagement (1). The repetitive and cyclical action of gait is dynamically modulated in real-time according to an individual’s target speed, a process integrating neuromuscular coordination, biomechanical adjustments, and sensory feedback to optimize locomotor efficiency and adaptability across diverse environmental contexts (2). Human gait may be conceptualized as a sequence of controlled incomplete falls in which the lower limbs function as inverted pendula, a biomechanical strategy that minimizes locomotor energy cost by reducing the vertical displacement of the body’s center of mass and optimizing gravitational energy transfer during bipedal locomotion (3, 4). The three-dimensional analysis of human gait has long been a focal point in biomechanics, clinical research, and interdisciplinary studies, encompassing advanced motion capture technologies, biomechanical modeling, and quantitative data interpretation to unravel the neuromuscular control mechanisms, pathological gait deviations, and adaptive locomotor strategies across diverse populations and environmental contexts (5). Three-dimensional gait analysis systems, integrating high-speed computer computation, force plates, and infrared high-speed cameras, enable the acquisition of lower limb kinematic data and electromyographic signals, thus facilitating the objective and quantitative characterization of subjects’ gait patterns and motor functions (6). Motion capture systems, the most widely applied gait analysis systems currently, translate the movements of body segments into three-dimensional digital data using optical or electromagnetic technologies, enabling precise quantification of locomotor patterns in biomechanical and clinical research (7). The specific approach entails placing markers at various anatomical positions on the subject, capturing motion data through cameras or electromagnetic receivers, digitizing the information to derive three-dimensional coordinate data, and integrating temporal parameters to compute the corresponding velocities and accelerations (Figure 1) (8).

Spinal disorders refer to pathological changes in the intervertebral discs, vertebrae, muscles, and ligaments, which can compress, traction, or irritate spinal nerves, the spinal cord, autonomic nerves, and blood vessels, thereby causing symptoms such as spinal pain, muscle strength and sensory impairment, limb radiating pain, and potentially leading to paralysis in severe cases (9–11). Studies have reported that the incidence of lumbar and cervical spondylosis in adults is as high as 60–80%, and even reaches 90% in groups such as workers, nurses, farmers, and truck drivers, highlighting the high morbidity of spinal diseases (12–15). Patients with spinal diseases often present with spinal degenerative lesions, and the body activates the spinal-pelvic-lower limb joint compensatory mechanism to maintain upright walking, thereby frequently leading to conditions such as pelvic tilt, gait abnormalities, and sagittal spinal-pelvic imbalance (16–18). Therefore, familiarity with and mastery of lower extremity functional status and gait in patients with spinal disorders are particularly crucial for guiding rational clinical diagnosis and treatment, as well as evaluating the efficacy of treatment and rehabilitation training in the later stage. Although gait abnormalities are highly prevalent among patients with spinal disorders, there is currently no clear quantitative assessment method in clinical practice, and traditional evaluation approaches often fail to fully capture the complex biomechanical characteristics and functions of patients’ gait. This is where three-dimensional (3D) gait analysis emerges as a transformative technology, offering precise movement data that can enhance the understanding of spinal disorders and inform personalized treatment strategies. By integrating advanced motion analysis techniques, clinicians can gain deeper insights into the biomechanical implications of spinal diseases, ultimately improving diagnostic accuracy, surgical planning, and rehabilitation outcomes for patients.

The application of 3D gait analysis in clinical settings allows for a comprehensive evaluation of gait dynamics, including spatiotemporal parameters, joint angles, and neuromuscular activity (19). This technology utilizes sensor technology to collect detailed movement data, which can be particularly beneficial for assessing the functional impairments associated with spinal diseases. Studies have shown that 3D gait analysis can effectively identify deviations in gait patterns among patients with lumbar degenerative conditions, providing valuable insights into their functional limitations and guiding therapeutic interventions (20). Moreover, the quantitative data obtained through this analysis can serve as a baseline for monitoring patient progress during rehabilitation, thereby facilitating tailored treatment plans that cater to individual needs. In surgical contexts, 3D gait analysis can play a pivotal role in preoperative planning by enabling surgeons to visualize how specific spinal conditions may affect a patient’s movement mechanics, thereby helping select the most appropriate surgical techniques and approaches and ultimately leading to improved surgical outcomes (20, 21). The correlation between gait parameters and clinical outcomes, such as pain levels and disability scores, has been demonstrated in various studies, indicating that enhanced understanding of gait dynamics can lead to more effective surgical interventions (22). Furthermore, post-surgical rehabilitation can be optimized by utilizing 3D gait analysis to track recovery progress and adapt rehabilitation protocols based on real-time feedback, ensuring that patients regain their functional capabilities as efficiently as possible. In summary, this review synthesizes the application of three-dimensional gait analysis in spinal disorders, with a specific emphasis on its critical significance in facilitating accurate diagnosis, optimizing surgical planning, and enhancing the effectiveness of rehabilitation processes.

## 2 Main body

### 2.1 3D gait analysis technology

Three-dimensional (3D) gait analysis technology has revolutionized the assessment of human locomotion by providing detailed insights into the mechanics of walking (23). This technology employs sophisticated motion capture systems that utilize multiple cameras and sensors to track the movement of markers placed on the body. The resulting data allows for a comprehensive analysis of gait patterns, including spatiotemporal parameters, joint angles, and forces exerted during walking. Such detailed assessments are crucial for diagnosing and monitoring various conditions, including neurological disorders, orthopedic injuries, and rehabilitative outcomes (20). The integration of 3D gait analysis in clinical settings has enhanced the precision of evaluations, enabling tailored rehabilitation programs that cater to individual patient needs (22).

#### 2.1.1 Technical principles and equipment

This technology relies on sophisticated motion capture systems, with multiple cameras strategically placed around the walking area to capture the movement of markers attached to specific anatomical landmarks on the body and working in tandem to track the position of the markers in three-dimensional space (23). In addition to cameras, sensors also play a crucial role. Inertial measurement units (IMUs), which measure acceleration, angular rate, and magnetic fields, can be used either alone or in combination with camera-based systems (24). IMUs are small, lightweight, and can be easily attached to the body, allowing for more flexible data collection, even in non-laboratory environments (24). Advanced systems may also incorporate electromyography (EMG) to assess muscle activity during gait (25). The accuracy of 3D gait analysis is significantly higher than traditional methods, making it the gold standard in clinical gait analysis (26).

#### 2.1.2 Data collection and processing methods

Data collection in 3D gait analysis involves a systematic approach where subjects walk along a designated path while their movements are recorded. The data collected from the motion capture systems is complex and requires sophisticated processing. The raw data from cameras and sensors is first pre-processed to remove noise and correct for any calibration errors. Algorithms are then applied to calculate various gait parameters. Spatiotemporal parameters, such as step length, stride length, gait velocity, and cadence, are relatively straightforward to calculate from the marker trajectories (27). Commonly used metrics include the Gait Deviation Index (GDI) and the Gait Profile Score (GPS), which provide a comprehensive overview of gait quality (28, 29). Additionally, machine learning algorithms are increasingly being integrated into data processing to enhance diagnostic accuracy and predict outcomes based on gait patterns (30). This innovative approach allows for the identification of subtle gait abnormalities that may not be evident through visual assessment alone (31).

#### 2.1.3 Comparison with traditional gait analysis

When compared to traditional gait analysis methods, 3D gait analysis offers several distinct advantages. Traditional methods often rely on subjective assessments and two-dimensional measurements, which can lead to inaccuracies and incomplete evaluations (32). In contrast, 3D gait analysis provides a quantitative, objective assessment of gait mechanics, allowing clinicians to visualize and analyze complex movements in three dimensions. This enhanced capability facilitates better diagnosis, treatment planning, and monitoring of rehabilitation progress (33). Furthermore, 3D gait analysis can capture a broader range of data, including kinematic and kinetic parameters, which are crucial for understanding the underlying causes of gait abnormalities (34). As a result, 3D gait analysis is increasingly recognized as a vital tool in both clinical and research settings.

### 2.2 Application of 3D gait analysis in the diagnosis of spinal diseases

**Figure 2:**
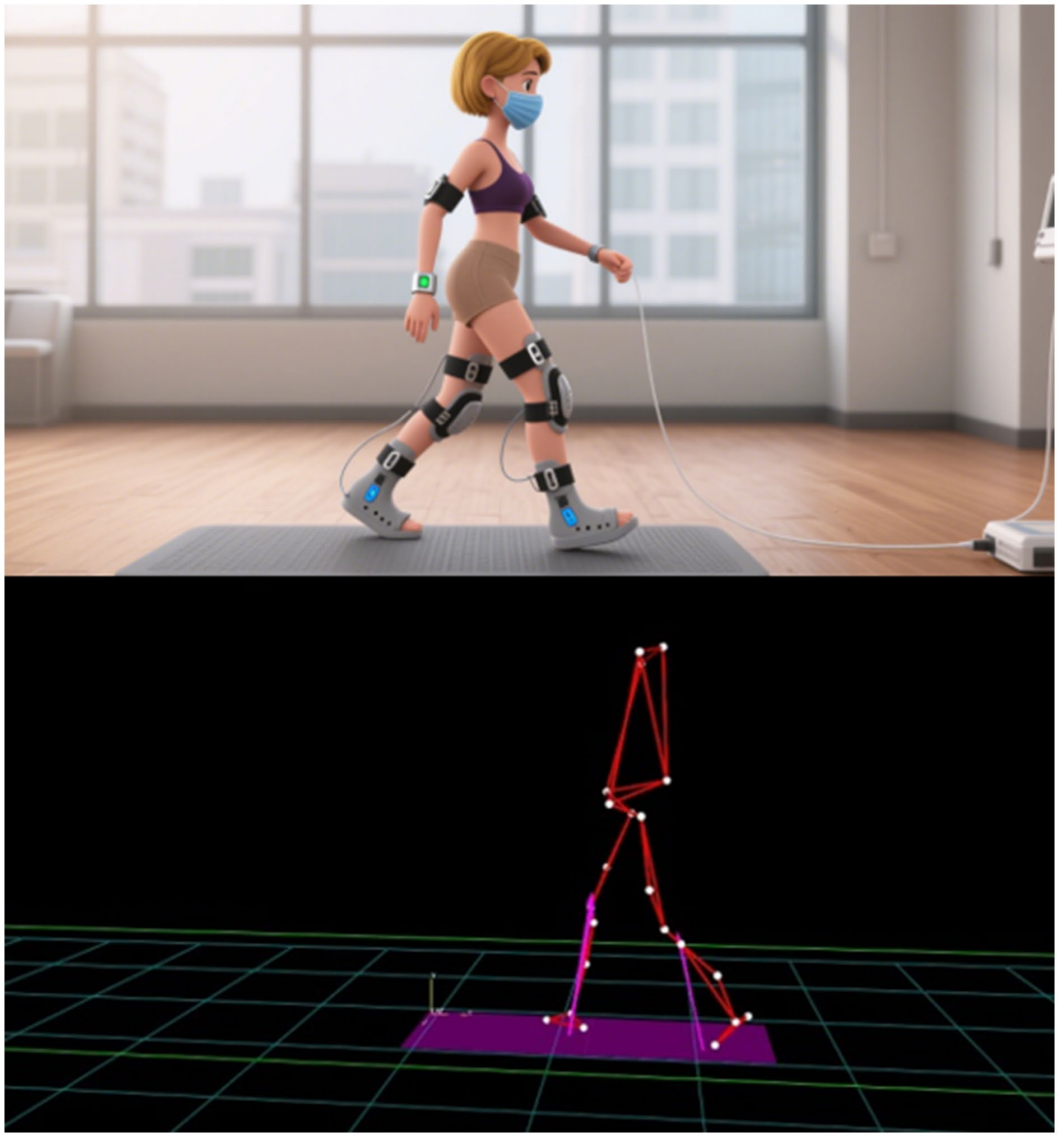
The three-dimensional gait analysis system quantitatively evaluates the gait of patients.

3D gait analysis has emerged as a significant tool in the diagnosis of spinal diseases, providing insights into biomechanical abnormalities that may not be apparent through traditional diagnostic methods (35). This technology utilizes advanced sensor technology to capture and analyze movement data, allowing for a detailed assessment of various gait parameters, including spatiotemporal metrics, joint angles, and neuromuscular activity (36). In patients with spinal conditions, such as lumbar degenerative diseases, 3D gait analysis can reveal subtle deviations in gait patterns that indicate underlying biomechanical dysfunctions (37). Studies have shown that patients with lumbar degenerative conditions exhibit altered gait characteristics, such as reduced gait speed and altered stride length, which can be quantitatively assessed through 3D motion analysis systems (Figure 2) (20). This capability to identify biomechanical abnormalities is crucial for clinicians aiming to develop targeted treatment plans and rehabilitation strategies for patients with spinal disorders.

#### 2.2.1 Identification of biomechanical abnormalities

The identification of biomechanical abnormalities through 3D gait analysis is pivotal in diagnosing spinal diseases. By capturing the intricate details of a patient’s gait, clinicians can detect deviations that may suggest underlying spinal pathologies (38). Patients with conditions like scoliosis or spinal stenosis often exhibit compensatory gait patterns that can be quantitatively analyzed using 3D motion capture technology (21). This analysis not only highlights deviations in joint angles and stride length but also provides insights into the distribution of forces across the lower extremities. Research has demonstrated that specific gait deviations, such as increased anterior pelvic tilt or altered knee flexion angles, are associated with various spinal disorders (22). By correlating these biomechanical markers with clinical symptoms, healthcare providers can enhance their diagnostic accuracy and tailor interventions to address the specific needs of each patient, ultimately improving outcomes in spinal disease management.

#### 2.2.2 Assessment of disease severity

3D gait analysis plays a crucial role in assessing the severity of spinal diseases by providing objective measurements that correlate with clinical findings. The ability to quantify gait parameters allows for a more nuanced understanding of how spinal conditions impact a patient’s functional mobility. For instance, studies have shown that patients with more severe lumbar degenerative diseases exhibit significant alterations in gait speed, stride length, and stance phase duration compared to those with milder forms of the disease (20). These quantitative assessments can be linked to established clinical scales, such as the Oswestry Disability Index (ODI) and the Visual Analog Scale (VAS) for pain, thereby offering a comprehensive view of the patient’s functional status (39–41). Furthermore, the data obtained from 3D gait analysis can serve as a baseline for monitoring disease progression over time, allowing clinicians to evaluate the effectiveness of therapeutic interventions and adjust treatment plans accordingly (42).

#### 2.2.3 Prediction of disease progression

The predictive capabilities of 3D gait analysis in spinal diseases are particularly valuable for anticipating disease progression and informing treatment strategies. By analyzing changes in gait patterns over time, clinicians can identify early signs of deterioration in patients with spinal conditions. Research indicates that alterations in gait speed and other kinematic parameters may precede clinical symptoms, providing a window of opportunity for early intervention (43). This proactive approach is essential in managing progressive spinal disorders, as timely interventions can mitigate further degeneration and improve quality of life. Moreover, the integration of 3D gait analysis with other diagnostic modalities, such as imaging studies, enhances the overall understanding of a patient’s condition, facilitating a more comprehensive approach to predicting and managing the progression of spinal diseases (44). By leveraging the insights gained from gait analysis, healthcare professionals can implement targeted rehabilitation programs that address specific biomechanical deficits, ultimately leading to better patient outcomes.

### 2.3 The role of 3D gait analysis in surgical planning

Three-dimensional (3D) gait analysis has emerged as a pivotal tool in the realm of surgical planning, particularly in orthopedic and neurosurgical procedures (45, 46). By providing a detailed assessment of a patient’s gait mechanics, clinicians can tailor surgical interventions to address specific biomechanical deficits. This personalized approach not only enhances the precision of surgical strategies but also aligns treatment plans with the unique anatomical and functional needs of each patient. The integration of 3D gait analysis into preoperative assessments allows for an in-depth understanding of a patient’s movement patterns, which is invaluable for optimizing surgical outcomes and minimizing postoperative complications (47). Moreover, this technology facilitates the identification of underlying gait abnormalities that may not be apparent through traditional assessment methods, thereby informing more effective surgical strategies and rehabilitation protocols.

#### 2.3.1 Development of personalized surgical strategies

The utilization of 3D gait analysis in surgical planning enables the creation of personalized surgical strategies that are specifically designed to meet the individual needs of patients. By analyzing various gait parameters, such as stride length, cadence, and joint angles, surgeons can identify specific deficits and tailor their approach accordingly (20). For instance, in patients with knee osteoarthritis, 3D gait analysis can reveal compensatory mechanisms that may influence surgical decisions, such as the choice between total knee arthroplasty versus partial knee replacement (48). Additionally, this analysis can help in predicting postoperative outcomes by correlating preoperative gait data with postoperative recovery trajectories. Studies have shown that personalized surgical strategies based on detailed gait analysis significantly improve functional outcomes and patient satisfaction post-surgery (43). This individualized approach not only enhances surgical precision but also fosters a more patient-centered care model, ultimately leading to better rehabilitation outcomes.

#### 2.3.2 Pre- and postoperative biomechanical assessment

The application of 3D gait analysis extends beyond surgical planning to encompass both pre- and postoperative biomechanical assessments (49). The application of 3D gait analysis extends beyond surgical planning to encompass both pre- and postoperative biomechanical assessments. Preoperatively, it serves as a baseline measurement that captures the patient’s functional status, allowing for a comprehensive evaluation of their gait mechanics. This information is crucial for setting realistic postoperative goals and expectations. Postoperatively, 3D gait analysis is instrumental in monitoring recovery and rehabilitation progress. By comparing pre- and postoperative gait data, clinicians can assess the effectiveness of the surgical intervention and make necessary adjustments to rehabilitation protocols. A study involving patients undergoing lumbar surgery demonstrated significant improvements in gait parameters postoperatively, indicating successful surgical outcomes and effective rehabilitation strategies (20). This continuous biomechanical assessment not only aids in tracking recovery but also provides valuable insights into the long-term effects of surgical interventions on gait dynamics (50).

#### 2.3.3 Case studies improving surgical success rates

Numerous case studies highlight the efficacy of 3D gait analysis in enhancing surgical success rates across various medical disciplines. In orthopedic surgery, the analysis has been used to refine techniques for joint replacements, leading to improved alignment and functionality post-surgery (51, 52). In one notable case, a patient with complex knee deformities underwent a personalized surgical intervention guided by 3D gait analysis, resulting in significant improvements in both gait mechanics and overall quality of life (22). Similarly, in neurosurgery, 3D gait analysis has been utilized to inform surgical strategies for patients with spinal deformities, demonstrating a marked reduction in postoperative complications and enhanced recovery times (53). These case studies underscore the transformative potential of integrating 3D gait analysis into surgical planning, ultimately leading to higher success rates and better patient outcomes across various surgical specialties.

### 2.4 Application of 3D gait analysis in rehabilitation

3D gait analysis has emerged as a vital tool in the rehabilitation field, enabling clinicians to assess and monitor patients’ movement patterns with high precision (54–56). This technology utilizes advanced sensor technology to collect comprehensive data on various gait parameters, including spatiotemporal characteristics, joint angles, and neuromuscular activity. The application of 3D gait analysis in rehabilitation holds particular significance, as it enables quantitative assessment of therapeutic intervention efficacy, facilitates data-driven optimization of treatment strategies tailored to individual patient profiles, and ultimately enhances functional outcomes by capturing nuanced spatiotemporal, kinematic, and kinetic parameters that reflect underlying locomotor impairments and recovery trajectories. By providing detailed insights into the biomechanics of walking, 3D gait analysis facilitates targeted rehabilitation approaches tailored to individual patient needs, ultimately leading to improved functional mobility and quality of life (57).

#### 2.4.1 Evaluation of rehabilitation outcomes

The evaluation of rehabilitation outcomes through 3D gait analysis is crucial for understanding the effectiveness of various therapeutic interventions. Research has demonstrated that this technology can significantly enhance the assessment of surgical outcomes and postoperative rehabilitation, particularly in patients with conditions such as lumbar degenerative diseases and stroke (37, 58). For instance, a study involving patients undergoing lumbar interbody fusion surgery found that 3D gait analysis provided valuable data correlating improvements in gait parameters with subjective measures of pain and disability, as indicated by the visual analog scale (VAS) and Oswestry Disability Index (ODI) scores (20). Furthermore, the ability to quantify changes in gait dynamics pre- and post-intervention allows clinicians to make informed decisions regarding the continuation or modification of rehabilitation programs. By establishing a clear link between objective gait metrics and patient-reported outcomes, 3D gait analysis serves as a reliable method for evaluating the success of rehabilitation strategies and identifying areas requiring further intervention.

#### 2.4.2 Optimization of exercise intervention programs

3D gait analysis plays a pivotal role in optimizing exercise intervention programs by providing precise measurements of gait mechanics that can inform rehabilitation strategies (59). By analyzing the kinematic and kinetic aspects of gait, clinicians can identify specific deficits in patients’ movement patterns, which can then be targeted through tailored exercise regimens (25). In patients recovering from stroke, 3D gait analysis has been shown to reveal compensatory movement patterns that may hinder recovery (60). By understanding these compensatory strategies, therapists can design interventions that not only focus on improving gait speed and stability but also address underlying neuromuscular control issues (61). Additionally, the integration of 3D gait analysis with wearable technology allows for real-time monitoring of patients’ progress during rehabilitation, enabling clinicians to adjust exercise prescriptions dynamically based on individual responses to treatment (58). This data-driven approach enhances the efficacy of rehabilitation programs, ensuring that interventions are both effective and personalized.

#### 2.4.3 Patient follow-up and long-term outcome monitoring

Long-term follow-up and monitoring of outcomes are essential components of effective rehabilitation, and 3D gait analysis provides a robust framework for assessing these aspects over time. Continuous monitoring of gait parameters enables clinicians to track changes in patients’ mobility and functional independence, which is particularly important for individuals with chronic conditions or those recovering from surgery. Studies have indicated that regular assessments using 3D gait analysis can identify subtle changes in gait mechanics that may signal the need for intervention or adjustment in rehabilitation strategies (22). Furthermore, the ability to correlate gait analysis data with clinical outcomes, such as quality of life and functional ability, enhances the understanding of the long-term effectiveness of rehabilitation interventions. This comprehensive approach not only aids in evaluating the success of rehabilitation but also informs future treatment planning, ensuring that patients receive ongoing support tailored to their evolving needs. By integrating 3D gait analysis into routine follow-up care, healthcare providers can significantly improve patient outcomes and enhance the overall quality of rehabilitation services.

#### 2.4.4 Connection with tele-rehabilitation protocols

Face-to-face physical therapy services can be directly provided to patients in their home environment, whereas telerehabilitation enables therapists to deliver rehabilitation from a remote location to a patient’s home (62). Although the COVID-19 pandemic strained health care systems globally, novel opportunities arose to expand and increase the use of in-home medical services such as therapy to provide personalized care and rehabilitation (63, 64). Gait analysis plays a critical role in the remote monitoring of rehabilitation for patients with orthopedic and neurological conditions (e.g., fractures, strokes, Parkinson’s disease) in telemedicine (65–67). While traditional 3D gait analysis relies on laboratory environments and is hampered by issues including high cost and low accessibility, existing technologies further struggle to simultaneously meet the requirements of accuracy, portability, real-time performance, and privacy compliance (68). For instance, mainstream human pose estimation (HPE) tools like OpenPose can ensure accuracy and real-time performance on high-performance computing platforms but fail to adapt to portable devices due to high resource consumption (69). In contrast, lightweight convolutional neural networks on mobile devices offer portability and privacy protection but lack sufficient accuracy for clinical applications (70–72). To address these technical challenges, Martini et al. proposed a portable 3D HPE platform named MAEVE (69). Built on low-cost hardware—including an RGB-D camera (e.g., Intel RealSense D415) and low-power embedded computing devices (e.g., NVIDIA Jetson series development boards)—the platform achieves a balance of performance through multi-stage optimizations (69). Martiš et al. evaluated the markerless smartphone-based technology, the OpenCap system, which, compared with marker-based systems, exhibited good accuracy in spatiotemporal parameters but still had certain limitations in capturing kinematic parameters (73). In summary, the role of gait analysis in telerehabilitation for a variety of orthopedic and neurological diseases has become increasingly critical in recent years; while its efficacy and role in telerehabilitation after surgery for spinal diseases have not yet been addressed in clinical studies, it still demonstrates substantial application potential.

### 2.5 The interplay between central nervous system injury and gait mechanics

The central nervous system (CNS), encompassing the brain and spinal cord, plays a pivotal role in orchestrating the complex process of gait (74, 75). Gait involves a highly coordinated sequence of muscle contractions and relaxations, which is precisely regulated by the CNS (76–78). However, when the CNS sustains an injury, whether from trauma, stroke, neurodegenerative diseases like Parkinson’s, or congenital conditions such as cerebral palsy, it can profoundly disrupt the normal gait pattern (79–84).

#### 2.5.1 Impact of CNS injury on gait mechanics

CNS injury often leads to a breakdown in the normal motor control mechanisms. In the case of spinal cord injury, for instance, the communication pathway between the brain and the muscles is severed or impaired (85). This disruption can cause muscle weakness, paralysis, or abnormal muscle tone. As a result, patients may experience difficulties in generating the appropriate forces for smooth locomotion. For example, they may have trouble initiating the swing phase of gait, leading to a delayed or jerky movement of the leg (86). In cerebral palsy, an injury to the developing brain, the motor control centers in the brain are affected (81, 87). This can result in spasticity, where muscles are constantly contracted, leading to abnormal postures and gait patterns like the characteristic scissor gait, where the legs cross over each other during walking (88, 89). The CNS is also responsible for integrating sensory information from various sources, such as proprioceptors in muscles and joints, and the vestibular system in the inner ear, to maintain balance and adjust gait in real-time (74). After a CNS injury, this sensory integration can be severely disrupted (74). Patients with stroke, for example, may experience hemispatial neglect, where they are unaware of one side of their body (90, 91). This lack of awareness can lead to imbalanced gait, as they may not properly weight-bear on the affected side (92, 93). In addition, damage to the proprioceptive pathways in the spinal cord can cause a loss of the sense of limb position (94, 95). Without this crucial feedback, individuals find it challenging to coordinate their movements accurately, leading to unsteady and often compensatory gait patterns (95–97). CNS injury can cause significant changes in the biomechanics of gait (80, 82, 84, 98). For example, in Parkinson’s disease, patients often exhibit a stooped posture and reduced arm swing, which affects the overall balance and gait efficiency (99, 100). The ground reaction forces, which are essential for normal walking, can also be altered (80, 101–103). A study on spinal cord - injured patients found that they had abnormal patterns of ground reaction forces, with reduced peak forces and altered force distribution during the gait cycle (80, 104, 105). These changes in biomechanics not only make walking less efficient but also increase the risk of falls and other secondary complications (80, 106, 107). Amyotrophic lateral sclerosis (ALS) is a multisystem neurodegenerative disease that encompasses cognitive and behavioral impairments (108). In ALS, cognitive impairments may be one of the factors contributing to motor function decline, and three-dimensional gait analysis in patients can assess disease progression, providing strong evidence for subsequent diagnosis and treatment (109, 110). Furthermore, this can inform the development of a bio-psycho-social model for gait assessment, thereby yielding broader implications for diagnosis and prognosis.

#### 2.5.2 Influence of altered gait on the CNS

The CNS has an inherent ability to adapt and reorganize, a phenomenon known as neural plasticity (111–114). When faced with gait abnormalities due to injury, the CNS attempts to compensate (115). For example, in the case of a stroke patient with hemiparesis (weakness on one side of the body), the unaffected side of the brain may start to take over some of the functions of the damaged area (116). This can be observed as the patient develops compensatory strategies during gait, such as using the non-affected arm more vigorously to maintain balance. However, these compensatory mechanisms are not always perfect and may lead to the development of new abnormal movement patterns over time (117). The abnormal gait patterns resulting from CNS injury can also have a feedback effect on the remaining neural circuits (86, 118). The continuous use of compensatory and inefficient gait patterns can lead to over-activation or under-activation of certain neuronal populations (119, 120). In spinal cord-injured patients, the repetitive abnormal loading and movement patterns can affect the spinal cord neurons, potentially leading to further neural damage or maladaptive changes (121, 122). This can create a vicious cycle, where the altered gait exacerbates the neural injury, which in turn worsens the gait problems. Understanding the intricate interplay between CNS injury and gait mechanics is crucial for developing effective rehabilitation strategies. Physical therapy, for example, can be designed to target specific gait abnormalities and promote neural plasticity (123). In the case of spinal cord-injured patients, locomotor training with body weight support can help to re-educate the neural circuits involved in gait by providing repetitive, task-specific movements (124). For patients with cerebral palsy, therapies such as constraint-induced movement therapy can be used to encourage the use of the affected limb and improve gait symmetry (125, 126). Additionally, emerging technologies like robotic exoskeletons and neuromuscular electrical stimulation can be incorporated into rehabilitation programs to assist with gait training and potentially enhance the recovery of normal gait patterns.

In conclusion, the relationship between CNS injury and gait mechanics is complex and bidirectional. CNS injury disrupts gait through motor control and sensory integration impairments, leading to altered biomechanics. Conversely, the resulting abnormal gait can impact the CNS, affecting neural plasticity and neuronal function. Further research in this area is essential to develop more targeted and effective therapeutic approaches for patients with CNS-related gait disorders.

### 2.6 The role of cognitive impairment in the alteration of gait patterns

Growing evidence indicates that cognitive impairment significantly alters gait patterns, with profound implications for early diagnosis and fall prevention in neurodegenerative diseases (127–129). This interaction between cognition and mobility reflects the disruption of a dynamic neural network involving the prefrontal cortex, hippocampus, and basal ganglia, which coordinate attention, executive function, and spatial navigation to maintain stable locomotion (110, 130). The quantitative relationship between cognitive decline and gait deterioration has been well-established through meta-analyses. A systematic review encompassing 29,520 participants demonstrated that gait speed decreases progressively across the cognitive spectrum: 0.11 m/s in mild cognitive impairment (MCI), 0.20 m/s in mild dementia, and 0.41 m/s in moderate dementia compared to cognitively healthy older adults (131). More notably, gait variability—characterized by irregular step timing and length—emerges as a sensitive marker, particularly under dual-task conditions where concurrent cognitive demands amplify motor deficits (132). This phenomenon is observed in both Alzheimer’s disease (AD) and Parkinson’s disease (PD), where dual-task-related gait changes precede traditional clinical symptoms by years (133, 134). Distinct gait signatures differentiate dementia subtypes, offering diagnostic value. The HUNT study revealed that individuals with AD perform better on physical assessments, including gait speed, than those with vascular dementia (VaD) or Lewy body dementia (LBD) (135). VaD and LBD patients exhibit more severe balance deficits and higher failure rates in functional tests like the five-times sit-to-stand, reflecting the vascular burden and cholinergic dysfunction specific to these subtypes (136). Recent innovations in assessment methodologies, such as curved path walking, have further enhanced early detection: 31 out of 50 gait markers show greater deviations in MCI during curve walking compared to straight paths, highlighting the utility of ecologically valid tasks (137). Neurological mechanisms underlying these changes involve impaired cognitive resource allocation. Executive dysfunction, particularly deficits in inhibition and task switching, disrupts the ability to adapt gait to environmental demands. Structural and functional alterations in the prefrontal cortex—critical for motor planning—correlate strongly with gait variability in MCI. Additionally, hippocampal atrophy impairs spatial orientation, contributing to navigational errors observed in curve walking tasks. These neurobiological changes transform gait from an automated process to a cognitively demanding task, increasing fall risk. Meta-analyses confirm that global cognitive impairment elevates serious fall-related injury risk by 2.13-fold, with executive function deficits independently associated with increased fall probability. Clinically, integrating gait analysis into cognitive assessments improves diagnostic accuracy. Low-cost videogrammetry systems can differentiate healthy elderly, MCI, and AD patients using parameters like gait velocity in 10-meter walks and dual-task timed up-and-go performance. The Montreal Cognitive Assessment (MoCA) demonstrates superior correlation with gait speed (*r* = 0.408) compared to the MMSE, particularly in detecting early cognitive decline. Such findings support the potential of gait parameters as accessible biomarkers for preclinical neurodegeneration.

In conclusion, cognitive impairment reshapes gait patterns through multifaceted neural disruptions, with subtype-specific manifestations. Advances in assessment technologies, from dual-task paradigms to curved path analysis, provide new avenues for early intervention. Recognizing gait alterations as both a consequence and marker of cognitive decline bridges neurology and geriatrics, offering opportunities to mitigate mobility loss and improve quality of life in aging populations.

### 2.7 Future research directions and technological advances

The field of gait analysis is undergoing rapid evolution, fueled by technological advancements and interdisciplinary collaboration. Future research will likely focus on integrating emerging technologies, fostering cross-disciplinary partnerships, and navigating the challenges and opportunities inherent in clinical translation. These directions are poised to enhance our understanding of human locomotion and drive impactful innovations in both research and practice.

#### 2.7.1 Application of emerging technologies in 3D gait analysis

Emerging technologies are set to revolutionize 3D gait analysis, enhancing its accuracy and applicability in clinical settings. Traditional gait analysis methods often rely on complex setups involving multiple cameras and markers, which can be resource-intensive and time-consuming. However, advancements in markerless motion capture systems, such as those utilizing depth cameras and artificial intelligence, are making gait analysis more accessible and practical (138). Systems like PathoOpenGait leverage 2D and 3D data from binocular cameras to monitor gait parameters, providing a cost-effective solution for continuous patient monitoring in clinical environments (139). Furthermore, wearable sensors and smartphone applications are emerging as viable alternatives, enabling real-time gait analysis in various settings, including home environments (140). These technologies not only reduce the burden on healthcare facilities but also allow for more personalized and timely interventions. Future studies should focus on validating these technologies against traditional methods to establish their reliability and accuracy in diverse patient populations.

#### 2.7.2 Potential for multidisciplinary collaboration

The future of gait analysis also hinges on the potential for multidisciplinary collaboration among healthcare professionals, engineers, and researchers. By combining expertise from fields such as biomechanics, neurology, rehabilitation, and computer science, innovative solutions can be developed to address complex gait disorders. And collaborative research has shown that integrating insights from neurophysiology with advanced motion capture technologies can lead to better understanding and treatment of gait impairments in conditions like Parkinson’s disease (141). Additionally, the development of standardized protocols for gait analysis across disciplines can enhance data comparability and improve clinical outcomes. Collaborative efforts can also facilitate the sharing of large datasets, which can be invaluable for machine learning applications aimed at predicting gait abnormalities and tailoring rehabilitation strategies. Encouraging interdisciplinary partnerships will be crucial in advancing the field and ensuring that gait analysis remains relevant and effective in clinical practice.

#### 2.7.3 Challenges and opportunities in clinical practice

As gait analysis technologies advance, they bring both challenges and opportunities for clinical practice. One of the primary challenges is the integration of new technologies into existing clinical workflows, which may require significant training and adaptation by healthcare professionals. Additionally, there is a need for rigorous validation of new tools to ensure they meet clinical standards for reliability and accuracy. For example, while wearable sensors and smartphone applications offer promising alternatives to traditional gait analysis, their clinical validation is still in progress (140). On the other hand, these challenges present opportunities for innovation in rehabilitation practices. The ability to conduct gait analysis in real-world settings can facilitate more personalized treatment plans and enhance patient engagement in their rehabilitation process. Moreover, the integration of artificial intelligence and machine learning into gait analysis can lead to more precise assessments and predictive analytics, ultimately improving patient outcomes. Addressing these challenges while capitalizing on the opportunities presented by technological advancements will be essential for the future of gait analysis in clinical practice.

## 3 Discussion

The advent of three-dimensional gait analysis (3DGA) has profoundly revolutionized the paradigms of diagnosing spinal disorders, formulating surgical intervention strategies, and designing rehabilitation protocols, thereby establishing itself as an indispensable quantitative tool in advancing clinical precision and patient outcomes within the field of spinal pathology (142–144). This cutting-edge technology facilitates a more holistic and nuanced comprehension of gait-related biomechanical aberrations, including spatiotemporal parameters, joint kinematics, and segmental dynamics, that directly informs evidence-based clinical decision-making processes by bridging the gap between qualitative observational assessments and quantitative functional metrics critical to optimizing therapeutic efficacy (145). By providing detailed data on the kinematics and kinetics of movement, 3DGA facilitates a more accurate assessment of the functional implications of spinal pathologies. This, in turn, enhances the precision of diagnosis and treatment planning, ultimately leading to improved patient outcomes.

Amidst the multifaceted challenges inherent to the diagnosis, management, and prognostication of spinal pathologies that encompass etiological heterogeneity, phenotypic variability, and dynamic functional interdependencies, there exists an urgent imperative to integrate interdisciplinary research perspectives including biomechanics, clinical neuroscience, rehabilitation science, and computational modeling to comprehensively unlock the transformative potential of 3DGA in advancing precision medicine, refining therapeutic algorithms, and enhancing long-term functional outcomes for affected patient populations. While biomechanical data derived from advanced technologies such as 3DGA remains invaluable for quantifying objective functional deficits and pathological movement patterns, equal emphasis must be placed on integrating clinical findings, validated patient-reported outcome measures (PROMs), and nuanced psychosocial contextual factors—including quality of life, socioeconomic barriers, and adaptive coping mechanisms—that collectively shape the lived experience of individuals with spinal disorders (146–148). By harmonizing these complementary research domains, clinicians and researchers can cultivate a more comprehensive, patient-centric understanding of how spinal pathologies permeate physical, psychological, and social dimensions of daily functioning, thereby laying the groundwork for the development of highly tailored, multimodal interventions that address both objective biomechanical aberrations and subjective patient needs to optimize treatment efficacy and long-term functional recovery.

Looking ahead, the prospects for 3DGA in the management of spinal diseases appear increasingly robust, driven by ongoing technological advancements that are enhancing its spatial–temporal resolution, reducing operational complexity, and improving real-time data processing capabilities. The expansion of 3DGA applications beyond conventional clinical environments—encompassing home-based monitoring, community rehabilitation settings, and workplace ergonomic assessments—promises to capture ecologically valid gait dynamics that more accurately reflect the impact of spinal pathologies on activities of daily living, instrumental tasks, and social participation, thereby filling critical gaps in our understanding of long-term functional trajectories and environmental modifiers of disease progression. Furthermore, the integration of 3DGA-derived biomechanical datasets with artificial intelligence and machine learning algorithms holds substantial potential for developing predictive analytics models that can identify preclinical gait biomarkers, stratify patient risk for disease exacerbation, and generate personalized treatment algorithms—incorporating timing of interventions, intensity of rehabilitative cues, and adjustment of surgical parameters—that optimize preventative strategies and therapeutic precision, ultimately moving the field toward a more proactive, individualized paradigm of spinal disease care that prioritizes functional preservation and quality of life.

Although 3DGA has demonstrated extremely strong potential, there are still challenges in the transformation between technical capabilities and clinical practicality. Technical constraints form a primary barrier. Marker displacement due to soft tissue artifacts remains problematic, particularly in scoliosis patients with abnormal trunk mechanics, compromising kinematic accuracy (149, 150). Traditional marker sets often treat the spine as a rigid structure, failing to capture segmental dynamics critical for spinal pathology evaluation (151, 152). While 3DGA shows high reliability for most lower limb parameters in spinal cord injury (SCI) and chronic low back pain, hip rotation assessment demonstrates concerning variability, with intraclass correlation coefficients below clinical acceptability thresholds (153). These technical inconsistencies undermine confidence in longitudinal monitoring. Clinical implementation hurdles further impede adoption. Patient-specific factors, including pain-related gait modifications in lumbar spinal canal stenosis (LSS) and limited endurance in severe spinal deformities, introduce measurement biases (154, 155). The lack of disease-specific assessment protocols exacerbates this issue—gait parameters validated for SCI may not apply to degenerative spinal conditions, creating a “one-size-fits-all” dilemma (44, 156). Post-measurement, the interpretive complexity of 3DGA datasets presents another obstacle; the sheer volume of kinematic and kinetic parameters requires specialized expertise rarely available in general clinical settings (143). Economic and logistical barriers remain underappreciated. Traditional 3DGA systems demand significant capital investment and technical maintenance, restricting access to specialized centers (154). While wearable inertial sensors offer cost-effective alternatives, their validity in spinal disease populations remains insufficiently validated compared to gold-standard motion capture (157). The absence of standardized databases and reference values for spinal disease subtypes further hinders widespread implementation. Addressing these limitations requires a dual approach: developing spine-specific marker sets and analysis models, alongside establishing disease-specific reference standards. Future advancements must balance technical precision with clinical pragmatism, potentially integrating artificial intelligence to streamline data interpretation. Until these obstacles are overcome, 3DGA will remain underutilized in routine spinal disease management.

In conclusion, the progress made in 3D gait analysis represents a significant leap forward in spinal disease management. As we strive to improve clinical outcomes, it is essential to foster collaboration among researchers, clinicians, and patients to ensure that this technology is utilized to its fullest potential. By embracing a multifaceted approach that respects both the quantitative data provided by 3DGA and the qualitative experiences of patients, we can advance the field of spinal health and ultimately enhance the quality of care provided to those affected by these challenging conditions.
